# Editorial: Emerging research on social security and population health: new opportunities and challenges

**DOI:** 10.3389/fpubh.2024.1412679

**Published:** 2024-06-04

**Authors:** Weike Zhang, Shangfeng Tang, Bishwajit Ghose

**Affiliations:** ^1^School of Public Administration, Sichuan University, Chengdu, China; ^2^School of Medicine and Health Management, Huazhong University of Science and Technology, Wuhan, China; ^3^Interdisciplinary School of Health Sciences, University of Ottawa, Ottawa, ON Canada

**Keywords:** social security, population health, health expenditures, economic uncertainty, digital economy, new rural social pension insurance

Academics have extensively explored the relationship between social security and population health, considering various aspects such as the mechanisms and economic implications of social security on health, as well as the influence of population health on the sustainability of social security. Recent years have witnessed the emergence of new economic and social dynamics, such as the rise of the digital economy, the economic downturn caused by the COVID-19 pandemic, and the acceleration of population aging. These factors have introduced both opportunities and challenges to the interplay between social security and population health. For instance, advancements in digital medical technology have enhanced the efficiency and health outcomes of medical insurance operations, whereas the aging population poses health risks to the older adult and increases their healthcare expenses by potentially reducing pension benefits. The complexity of these environmental changes complicates the understanding of the relationship between social security and population health, necessitating further research for analysis.

This Research Topic aims to gather original qualitative and/or quantitative research articles that deepen our comprehension of the relationship between social security and population health within the context of evolving social and economic conditions. The call for articles specifically focuses on exploring the opportunities and challenges presented by social security in influencing population health under new environmental circumstances. Additionally, contributions examining the connection between social security and population health from novel perspectives, including the underlying mechanisms and economic implications of social security's impact on population health, are encouraged. In sum, any original and significant research concerning social security and population health is of interest for this Research Topic.

In this editorial, we provide a summary of the articles published in the Research Topic “*Emerging research on social security and population health: new opportunities and challenges*” in the journal Frontiers in Public Health. Li and Chen identify a relative poverty standard based on medical needs, examines the impact of public health expenditures on medical expenses across various household types, and assesses the influence of public health spending on meeting the medical needs of households in relative poverty. Their findings reveal a crowding-out effect of public health expenditure on household medical spending, particularly pronounced in relatively poor households. Bocean and Vărzaru explore the association between social protection and health outcomes in EU countries using structural equation modeling (SEM) and cluster analysis. Their results demonstrate a significant positive relationship between social protection spending in EU countries and healthcare status. Increased social protection expenditure is linked to enhanced access to healthcare services and facilities.

Liu utilize a Bayesian panel vector autoregressive model to investigate the impact of economic uncertainty on public health. The analysis is based on an annual country-level panel dataset comprising 103 emerging markets and developing countries, covering the period from 1995 to 2019. Their results indicate that while the immediate effects of increased economic uncertainty on health appear to be minimal, it may lead to a longer life expectancy and reduced mortality rates over time. In a study conducted by Zhou et al., the relationship between the digital economy and residents' health is explored using data from the China Family Panel Studies (CFPS) in 2020. Their findings suggest that the digital economy has significantly enhanced the overall health status of residents, particularly those residing in the eastern region. The positive impact of the digital economy on residents' health primarily stems from its promotion of regional green development.

Song et al. utilize a multivariate ordered logistic regression model to investigate the impact mechanism and effects of the new rural social pension insurance (NRSPI) on the health of the older adult population in rural China. Based on survey data from the China Health and Retirement Longitudinal Study conducted in 2015 and 2018, their findings indicate that participation in the NRSPI leads to a significant improvement in the health of the rural older adult population. Moreover, the results of the heterogeneity analysis demonstrate that the NRSPI has a notable impact on the self-reported health of older adult individuals in rural areas, as well as a positive effect on both the mental and physical health of older adult women in rural regions.

Pu et al. explore the impact of the urban–rural resident basic medical insurance (URRBMI) on physical health of the rural older adult in China, utilizing data from the Chinese Longitudinal Healthy Longevity Survey (CLHLS) data in 2018. Their study reveals that URRBMI has a substantial positive impact on the physical health of rural older adults, particularly those in the eastern regions and those who are more advanced in age. In a separate study, Li and Yuan analyze data from the China Health and Retirement Longitudinal Study (CHARLS) spanning from 2011 to 2018, employing a staggered difference-in-differences model to evaluate the effects of integrating urban-rural health insurance on poverty vulnerability among rural residents. Their results indicate a significant reduction of 6.32% in poverty vulnerability due to the integration of urban-rural health insurance. Furthermore, the analysis of heterogeneity demonstrates that the integration of urban-rural medical insurance has a more pronounced impact on vulnerable groups with poorer health conditions than on those with better health, leading to a significant decrease in poverty vulnerability among individuals with chronic diseases.

Fan and Hua investigate the spillover effects and influencing mechanisms of the new rural insurance policy on human capital investments in rural households using data from the China Family Panel Studies (CFPS) in 2010, 2012, 2014, 2016, and 2018. The findings indicate that participation in the New Cooperative Medical Scheme (NCMS) significantly boosts human capital investments in rural households. Additionally, the spillover effects of the new policy vary significantly based on the gender, insurance stage, and family income of the insured individuals. The new rural insurance policy influences human capital investments in rural households through intergenerational interactions, impacting both the material aspects such as economic support, housework, and child care, as well as the non-material aspects like pension awareness.

## Author contributions

WZ: Writing – original draft. ST: Writing – review & editing. BG: Writing – review & editing.

